# SG-APSIC1024: The importance of the washing evaluation of flusher disinfector in the medical site: Visual evaluation with the ISO standardized test soil and adenosine triphosphate level

**DOI:** 10.1017/ash.2023.103

**Published:** 2023-03-16

**Authors:** Mari Shima, Rika Yoshida, Takashi Okubo

**Affiliations:** Former Employee of Tokyo Medical and Dental University Hospital, Division of Infection Control and Prevention, Japan; Division of Infection Prevention and Control, Tokyo Healthcare University Postgraduate School, Faculty of Healthcare, Department of Healthcare, Tokyo, Japan; Division of Infection Prevention and Control, Tokyo Healthcare University Postgraduate School, Faculty of Healthcare, Department of Healthcare, Tokyo, Japan

## Abstract

**Objectives:** We examined the washing evaluation method of the flusher disinfector installed in the medical site by visual evaluation using an ISO test soil and adenosine triphosphate (ATP) measurement. **Methods:** The test soil shown in ISO15883-5 was applied to the bedpan using 2 methods (N = 10) and was then visually evaluated using a 4-step scale. The ATP value on the surface of the bedpan corresponding to each scale was measured, and the correlation with the visual scale was confirmed. In addition, a visual evaluation was performed using a different flusher disinfector and bedpan (N = 3). **Results:** In the visual evaluation, when the test soil was applied to the entire surface of the bedpan, it remained in the parts where the water flow was hard to hit. When the test soil was applied to the surface of the bedpan except the lid, no soil remained. The 4-step visual evaluation scale and the logarithm of ATP value showed a positive correlation. In visual evaluations with different flusher disinfector and bedpan combinations, the residual soil patterns tended to be different. Some bedpans showed washing failure related to improper design. **Conclusions:** Poor cleaning of flusher disinfectors may occur when the amount of water used is insufficient or when the bedpan is significantly contaminated. It is important to carry out flusher disinfector washing evaluation at medical sites to evaluate the function of flusher disinfectors. An appropriate program must be utilized for staff education and appropriate management. Because flusher disinfectors and bedpans differ, flusher disinfector cleaning evaluations should be carried out at all facilities.

